# Analysis of Bee Population Decline in Lombardy during the Period 2014–2016 and Identification of High-Risk Areas

**DOI:** 10.3390/pathogens10081004

**Published:** 2021-08-09

**Authors:** Veronica Cappa, Monica Pierangela Cerioli, Alessandra Scaburri, Marco Tironi, Marco Farioli, Claudia Nassuato, Silvia Bellini

**Affiliations:** 1 Istituto Zooprofilattico Sperimentale della Lombardia ed Emilia-Romagna, Via Bianchi 9, 25124 Brescia, Italy; monicapierangela.cerioli@izsler.it (M.P.C.); alessandra.scaburri@izsler.it (A.S.); marco.tironi@izsler.it (M.T.); silvia.bellini@izsler.it (S.B.); 2 Direzione Generale Welfare di Regione Lombardia, Unità Organizzativa Veterinaria, Piazza Città di Lombardia, 20124 Milan, Italy; Marco_Farioli@regione.lombardia.it (M.F.); claudia_nassuato@regione.lombardia.it (C.N.)

**Keywords:** bees, population decline, plant protection, soil, risk, GIS

## Abstract

The first events of bee decline in Italy were reported during 1999. Since then, population decline has frequently been reported in Lombardy. In this study, the association between bee decline and the type of land surrounding the apiary was evaluated. A risk map was developed to identify areas with the highest risk of decline. Apiaries in Lombardy were selected from the national beekeeping database (BDA). The study period was from 2014 to 2016. Apiaries were deemed “declined” if they reported at least one event of decline or tested positive for plant protection products; apiaries were “not declined” if they did not report any events of bee decline during the study period. Out of 14,188 apiaries extracted from the BDA, 80 were considered declined. The probability of an apiary being declined increases by 10% in orchards and by 2% in arable land for each additional km^2^ of land occupied by these crops. The study showed an association between bee decline and the type of territory surrounding the apiaries, and the areas at the greatest risk of decline in Lombardy were identified. This information can be used by Veterinary Services as a predictive parameter for planning prevention and control activities.

## 1. Introduction

According to the International Union for Conservation of Nature (IUCN), more than 40% of the invertebrate species that are responsible for pollination, especially bees and butterflies, are at risk of disappearing. In particular, in Europe, 9.2% of bee species are currently threatened with extinction [1]. The Food and Agriculture Organization of the United Nations (FAO) estimates that of the 100 crops that provide 90% of the world’s food, 71 are pollinated by bees. Pollinators, therefore, play a key role in regulating the processes that sustain food production, habitat conservation, and natural resources, and are thus also fundamental to the conservation of biological diversity, the basis of economies, and very existence [2].

In Italy, the first reports from beekeepers on bee deaths and declining populations date back to 1999 and increased sharply in 2008 [3,4]. The causes of decline in population are manifold and involve a number of factors including climate change, the presence of pathogens, air pollution, habitat changes with a decrease in melliferous plants, intensive agriculture, and the use of plant protection products [5,6,7]. Furthermore, according to Porrini et al. [8] and Martinello et al. [9], the cause of decline in population can be defined based on the period in which it occurs. If the die-off occurs in the spring–summer, in intensively farmed areas, and during the period of sowing coated maize seed, weeding wheat, and treating fruit trees, the cause is often attributable to the use of plant protection products. On the other hand, if damage is detected between late summer and the end of the following winter, population decline is mainly due to diseases affecting the bees and the hive.

A study carried out in Italy in the period 2011–2014 to establish the state of health of bees and identify the possible causes of population decline collected data on the health of bees from 63 apiaries located throughout the country [6]. The study found that, during the period under study, 241 samples of bee matrices collected following reports of mortality in Italy tested positive for pesticides. Among the highly toxic insecticides, tests were positive for neonicotinoids (19%), pyrethroids (18%), and organophosphorus (16%).

In Lombardy, a worrying decrease in the number of hives and apiaries had already been observed in the period 2008–2009, and, in 2008, population decline was reported in connection with the maize sowing period. Following these reports, the Veterinary Services of the Lombardy Region coordinated the monitoring of reports by taking samples of bees and sending them to the laboratory for further analysis on the basis of the suspicions. The purpose of the above-mentioned monitoring was to acquire data and information both on the state of health of the regional bee population, and on the possible causes of population decline.

This is the context for this study, which seeks to determine whether there is an association between the decline in bee population and the types of crops surrounding the apiary. In addition, risk maps were created and areas at high risk of depopulation were identified. The data analysed refer to occurrence of population decline recorded in Lombardy from 2014 to 2016.

## 2. Results

For this study, a total of 14,188 apiaries from 5646 farms in Lombardy were analysed in the period 2014 to 2016.

The provinces with the highest number of apiaries are Bergamo (2125) and Brescia (2482); those with the highest percentage of population decline are Mantua (1.07%), Sondrio (0.94%), Brescia (0.85%), Lodi (0.83%), Milan (0.83%), Cremona (0.63%), and Pavia (0.51%) (Table 1).

Of the 14,188 apiaries in Lombardy in the period 2014 to 2016, 80 apiaries (0.56%) reported at least one event of population decline or positive results from tests for plant protection products; of these, 75 apiaries experienced population decline only once in the three-year period, and five apiaries reported it twice (Table 2).

Table 3 shows the month in which the decline in population was reported for each year considered. Population decline occurred in the spring (April–May) in 64% of cases, which is the period in which maize and orchards are treated.

Table 4 shows the type of land surrounding the apiaries (within 1.5 km). We can see that 93.4% of the apiaries without population decline have at least one wooded area next to them, compared with 88.8% of the apiaries that were declined. There are bushes close to 97.9% of the apiaries that were not declined and 98.8% of the apiaries that did see decline in population. Apiaries with population decline were more frequently located near arable land (93.8% compared to 89.6% of apiaries with no decline), orchards (61.3% compared to 54.8% of apiaries with no decline) and vineyards (52.5% compared to 45.8% of apiaries with no decline).

Table 5 shows the percentage of land occupied by the different types of land surrounding the apiaries by province. Woodland is prevalent in the province of Como (64.3%), Lecco (62.8%), Sondrio (62.8%), and Varese (69.7%), while apiaries in the provinces of Cremona (0.7%), Lodi (2.4%), and Mantua (2.0%) are located far from these types of land. Sondrio is the province with the highest percentage of orchards (3.1%), followed by Mantua (1.6%). Apiaries located in the provinces of Cremona and Mantua are mostly located close to arable land (82.8% and 82.0%, respectively). Finally, 9.9% of Pavia’s apiaries are located near vineyards, followed by the provinces of Brescia (4.2%) and Sondrio (3.3%).

Table 6 shows that the risk of population decline is associated with the size of the type of land surrounding the apiary (within 1.5 km). In other words, the larger the area of a given type of land, the greater the probability of a decline in population. In particular, the probability of an apiary declining in population statistically increases by 10% (OR: 1.10 95%CI (1.05;1.14), *p* < 0.0001) in the presence of orchards, and 2% of 1.02 (95%CI (1.01;1.04), *p* = 0.003) in the presence of arable land, for every additional km^2^ of land. This means that the risk of population decline increases with the unit increase (1 km^2^) of the surrounding territory. Furthermore, the average area occupied by these types of land is 0.20 km^2^ and 2.67 km^2^ for apiaries with population decline, and 0.06 km^2^ and 1.98 km^2^ for those without population decline, respectively.

**Figure 1:**
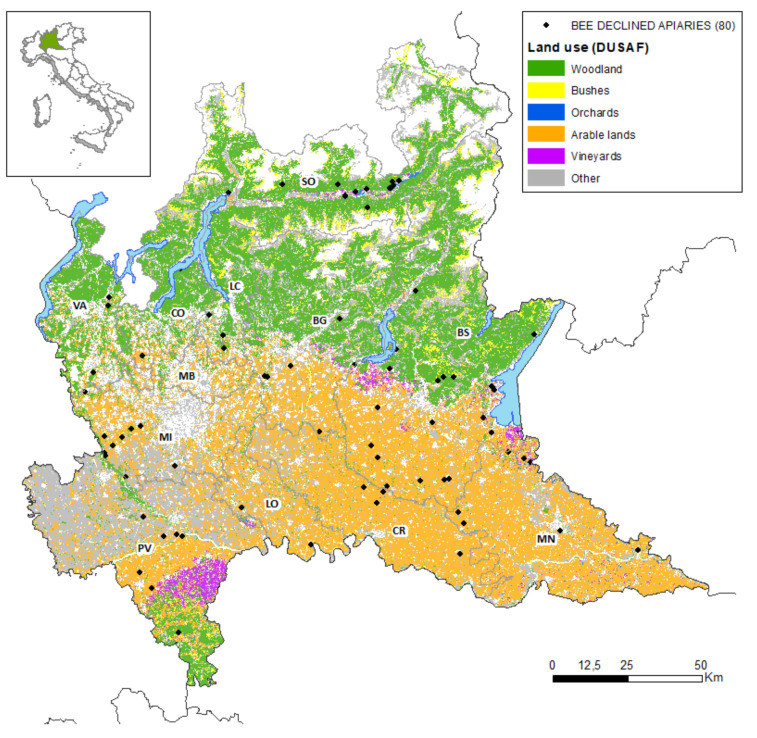
Map of land use (DUSAF 2015) and apiaries where population decline events have been recorded. Provinces: BG = Bergamo, BS = Brescia, CO = Como, CR = Cremona, LC = Lecco, LO = Lodi, MB = Monza Brianza, MI = Milan, MN = Mantua, PV = Pavia, SO = Sondrio, VA = Varese.

**Figure 2:**
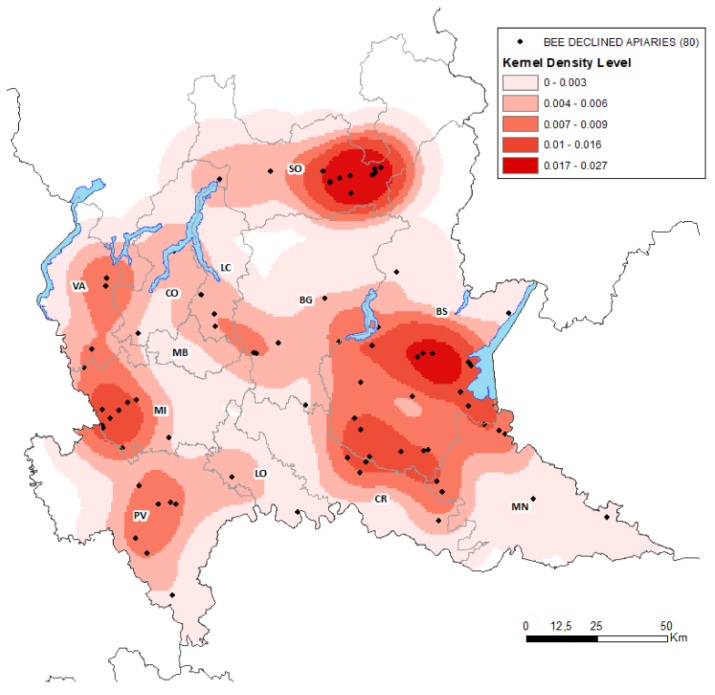
Lombardy with areas at risk of population decline. The density was estimated by means of the Kernel algorithm using the Epanechinikov distribution with bandwidth of 10 km; Provinces: BG = Bergamo, BS = Brescia, CO = Como, CR = Cremona, LC = Lecco, LO = Lodi, MB = Monza Brianza, MI = Milan, MN = Mantua, PV = Pavia, SO = Sondrio, VA = Varese.

Figure 1 shows the apiaries with population decline in Lombardy in relation to the type of land, while Figure 2—via the density estimated using the Kernel algorithm—shows the regional areas most at risk of population decline (in red), where the color intensity denotes the degree of risk; the more intense the color, the more the area is at risk of population decline. The map shows that declined apiaries are often located near areas rich in orchards (as in the case of the province of Sondrio), areas rich in orchards and arable land (as in the lower Garda area), and areas rich in arable land (as in the west, in the province of Milan, and on the border between the provinces of Brescia and Cremona).

## 3. Discussion

It has been amply demonstrated that the treatment of arable land, which leads to the dispersion of dust containing insecticide, is among the major contributors to the decline in bee population, supporting data found in other studies [7,9]. However, the quantification of the risk associated with the type of crop remains unknown.

This study aimed to quantify the risk of population decline by identifying the type of land surrounding the apiary according to the bee’s flight radius and a statistically significant association emerged between arable land and bee deaths. The risk was quantified considering the area occupied by type of land near the apiary, and the study shows a statistically significant association between orchards and bee deaths. The mortality recorded in apiaries near fruit orchards was traced to treatments carried out on fruit trees, as they are susceptible to insect and aphid attacks and plant protection products are widely used to combat these infestations.

The results of this study are in line with those obtained in the study conducted by Porrini in 2016, where a statistically significant linear correlation was demonstrated between the percentage of land area used for agriculture and the mortality rate of the colonies [8,10]. However, in Porrini’s study the authors did not distinguish between different type of crops.

Moreover, the timing of the population declines recorded in the Lombardy Region in the three-year period under consideration confirms the seasonality already reported in the literature [7,9]. Indeed, in this study it was observed that 64% of the population declines occurred in the spring (April–May), which is the preferred period for treating maize crops and orchards.

## 4. Methods

### 4.1. Data

The study area is the region of Lombardy in northern Italy, which includes three distinct natural zones: mountains, hills, and plains. Data processing was carried out using the following databases: (i) the national Beekeeping Database (BDA) [11]; (ii) reports of population decline from 2014 to 2016; (iii) the Agricultural and Forest Land Use (DUSAF) land use database; (iv) the lab results database from the Istituto Zooprofilattico Sperimentale della Lombardia e dell’Emilia Romagna (IZSLER).

#### 4.1.1. Information on Apiaries

The data on population decline were collected from the report form that beekeepers filled in during the period from 2014 to 2016 for the Veterinary Services, from the IZSLER information system that collects the data and the results of the sample surveys carried out.

The following information was collected through these forms: details of the beekeeper and the apiary affected by population decline, information on health, territory, and date of observation of the population decline, information on the bees with an indication of the number of hives affected, indicative number of dead bees and behavior of surviving bees, and information provided by the beekeeper on the possible cause of the damage linked to treatments (type of treatment, crop treated, and type of product used).

For this study, we only considered apiaries declined following suspected use of plant protection products or in which, in the absence of a specific report, the active ingredients used for crop treatments were identified. Apiaries in which an infectious disease or infestation was suspected, or where only bacterial/viral/parasitic agents were detected, were excluded.

#### 4.1.2. Agricultural and Forest Land Use Data

The Lombardy Region publishes the DUSAF land use data. This database collects information on the main types of land throughout the region and georeferences them. The last update was performed in 2018 using AGEA aerial photographs and satellite imagery. Thanks to this data source, it was possible to identify the agricultural use of the land surrounding the apiaries as well as the relative area of land occupied (in km^2^) out of the total area covered by the bees.

The types of land included in the analyses are described in Table 7, while areas of human activity, wetlands, and water bodies were excluded. Rice fields, arboriculture for timber production (poplar groves and other agricultural wood species), permanent grassland, natural high-altitude grassland, and olive groves were grouped in the category “Other” because they are poorly represented if considered separately.

### 4.2. Statistical Analysis

The Lombardy apiaries were divided into two groups: (i) apiaries were “bee decline” if in the three-year period 2014–2016 they reported at least one event of decline in bee population or tested positive for plant protection products, (ii) apiaries were “no bee decline” if in the three-year period 2014–2016 they did not report any decline in bee population. In the study, all apiaries for which a report of population decline was received from the beekeeper were considered declined, regardless of the laboratory test results, as chemical analyses are often unable to detect the toxic substance responsible for population decline. It is also difficult to detect chemical contamination because most bees affected by plant protection products do not return to the hive [3].

The characteristics of the farms and apiaries by population decline group (yes/no) were described using absolute frequencies and percentages.

The association between cases of population decline and the area occupied (in km^2^) by each DUSAF macro-category was estimated through the logistic regression model, considering the population decline group as the dichotomous dependent variable and the area occupied by each type of land as the independent variable (woodland, bushes, orchards, arable land, vineyards, and others). Considering that a study conducted in 1984 by Crane [12] estimated the flight radius of a forager bee to be about 1500 metres (an estimated area of 7.065 km^2^ around the apiary), we were able to use the georeferencing of the regional territory to identify for each apiary the types of crops located within this area.

All statistical analyses considered a threshold of statistical significance of 5%.

Finally, we mapped the density of population decline, calculated using Kernel’s methodology [13], a spatial interpolation technique that identifies the areas at greatest risk of population decline. The density estimate was calculated using the Epanechinikov distribution and a bandwidth (h) of 10 km, with the red scale identifying areas at risk of population decline: the more intense the color, the greater the risk.

### 4.3. Software Used

The statistical analysis was performed using the software R version 3.6.1 [14]. The maps were created using the software QGIS Development Team [15].

## 5. Conclusions

Bees are considered indicators of the health of the environment in which they live and exposure to certain insecticides or other toxic substances used during the flowering period can lead to major population decline. In order to standardise monitoring activities following reports of a population decline, the national *Guidelines for the management of reports of hive deaths and population declines related to the use of crop protection products* were issued [16]. The purpose of these guidelines is to ensure coordinated, rapid, and effective interventions in response to reports. Protecting our bees is crucial for the protection of the heritage of beekeeping in terms of the role of bees as pollinating insects, but also for the food safety of beekeeping products. In fact, in addition to protecting beekeeping heritage, reports and the resulting control measures are also important for the protection of the end consumer. This study revealed a statistically significant association between bee population decline and arable fields and orchards, and we were able to quantify the risk according to the type of land surrounding the apiary. The analysis shows that the risk of bee decline increases statistically when it is located close to orchards and arable land. In the event of use of plant protection products, the active ingredients must be used in full compliance with the treatment instructions provided and, above all, users should be aware of the risks regarding toxicity to animals, specifically bees. Using the product on plants not in flower, as is sometimes recommended correctly, is not always enough to prevent the risks associated with using the product.

The timing of the population declines recorded in the Lombardy Region in the three-year period considered suggests that plant protection products are the cause; a result that would confirm the data in the literature. This means that the results of the territorial analysis, understood as the type of crops found around the apiaries and the correlated risk in terms of population decline, could be used as predictive parameters to be applied when planning prevention and control activities.

## Figures and Tables

**Table 1:** Provincial distribution of apiary bee population decline.

	No Bee Decline	With Bee Decline	%
**Province**			
Bergamo	2119	6	0.28%
Brescia	2461	21	0.85%
Como	1133	4	0.35%
Cremona	631	4	0.63%
Lecco	993	3	0.30%
Lodi	238	2	0.83%
Monza Brianza	544	0	0.00%
Milan	1077	9	0.83%
Mantua	742	8	1.07%
Pavia	1369	7	0.51%
Sondrio	1264	12	0.94%
Varese	1537	4	0.26%
Classification *			
Sedentary	11,273	68	
Nomadic	2703	5	
***missing data***	***132***	***7***	
***TOTAL***	***14,108***	***80***	

* Last update of the Beekeeping Database (BDA) 31 December 2020.

**Table 2:** Apiaries and bee decline in the region in the period 2014–2016.

	Year			BDA-Registered Apiaries in the Three-Year Period **
	2014	2015	2016	
Bee Decline *	33	25	27	80
No Bee Decline	7810	8970	11,014	14,108
Total	7843	8995	11,041	**14,188**

* among the apiaries recording population decline, 13 also tested positive for infectious diseases or infestations (American Plague, *Nosema* spp., DWV and *Varroa* spp.). ** apiaries that were recorded at least once in the three-year period.

**Table 3:** Month of reported decline in population.

	Year			Total
	2014	2015	2016	
January	0	0	1	1
February	1	0	0	1
March	2	3	0	5
April	16	6	7	29
May	6	8	11	25
June	1	2	5	8
July	1	3	2	6
August	4	0	0	4
September	1	0	0	1
October	0	0	1	1
November	1	0	0	1
December	0	1	0	1
Total	33	23	27	83 *

* for 2 apiaries, the month in which population decline occurred is not available.

**Table 4:** Number and proportion of apiaries occupying different types of land.

	No Bee Decline n (%)	Bee Decline n (%)
Total number of apiaries	14,108	80
Number (%) of locations near *:		
Woodland	13,171 (93.4%)	71 (88.8%)
Bushes	13,815 (97.9%)	79 (98.8%)
Orchards	7734 (54.8%)	49 (61.3%)
Arable land	12,641 (89.6%)	75 (93.8%)
Vineyards	6459 (45.8%)	42 (52.5%)
Other	14,068 (99.7%)	79 (98.8%)

* the percentages were calculated considering the denominator of 14,108 apiaries with no population decline and 80 with population decline.

**Table 5:** Provincial distribution of apiaries by type of land occupied.

Province	Woodland	Bushes	Orchards	Arable Land	Vineyards	Other	Total
Bergamo	50.4%	3.1%	0.2%	25.1%	2.0%	19.2%	100%
Brescia	41.8%	3.7%	0.4%	34.6%	4.2%	15.3%	100%
Como	64.3%	2.5%	0.1%	14.2%	0.2%	18.9%	100%
Cremona	0.7%	1.3%	0.1%	82.8%	0.03%	15.0%	100%
Lecco	62.8%	3.0%	0.1%	14.1%	0.5%	19.4%	100%
Lodi	2.4%	1.7%	0.1%	75.4%	0.2%	20.2%	100%
Monza Brianza	24.2%	3.0%	0.2%	63.3%	0.3%	9.2%	100%
Milan	10.4%	2.7%	0.2%	66.6%	1.2%	19.0%	100%
Mantua	2.0%	1.3%	1.6%	82.0%	1.8%	11.3%	100%
Pavia	17.2%	5.1%	0.9%	39.7%	9.9%	27.1%	100%
Sondrio	62.8%	5.5%	3.1%	2.3%	3.3%	23.0%	100%
Varese	69.7%	2.0%	0.1%	19.6%	0.8%	8.5%	100%

**Table 6:** Association between population decline and the area occupied by types of land near the apiaries.

Type of Land	OR *	(95%CI)	*p*-Value	Average Area Occupied, with Population Decline (km^2^)	Average Area Occupied, No Population Decline (km^2^)
Forests	1.01	(0.99; 1.02)	0.32	1.84	2.24
Shrubland	1.05	(0.98; 1.10)	0.12	0.19	0.17
Orchards	1.10	(1.05; 1.14)	<0.0001	0.20	0.06
Arable land	1.02	(1.01; 1.04)	0.003	2.67	1.98
Vineyards	1.01	(0.98; 1.04)	0.40	0.33	0.31
Other	1.01	(0.99; 1.03)	0.41	0.84	0.92

* OR = odds ratio estimated via the logistic regression model.

**Table 7:** DUSAF Legend.

Macro-Category	DUSAF Type	Description
Woodland	Coniferous forests	Low, medium, and high-density coniferous forests
	Broadleaf forests	Low, medium, and high-density broadleaf forests, underbrush formations, chestnut groves
	Mixed broadleaf and coniferous forests	Low, medium, and high-density mixed coniferous and broadleaf forests
	Recent reforestation	Artificial forest systems not yet established and under treatment or to be treated. There are typically young trees with limited development of plants; generally, a regular planting pattern is recognizable. Trees are typically under 15 years of age. Plantations of poplars or other timber producing species included in another class are excluded
Bushes	Bushes and shrubs	Bushes, vegetation of riverbanks, vegetation of raised banks
	Developing areas	Bushes with significant presence of tall shrub and tree species, bushes in abandoned agricultural areas
Orchards	Orchards and berry farms	Plantations of non-rotational fruit trees that occupy the soil even for long periods and can be used for many years before being renewed.
Arable land	Simple arable land	Arable land, arboretums, horticultural crops, floricultural crops, family vegetable gardens, and arable land in irrigated areas
	Irrigated agricultural land	Irrigated agricultural land
Vineyards	Vineyards	Plantations of vines intended forthe production of both table and wine grapes
Other	Olive groves	Plantations of olive trees forproducing olives
	Arboriculture for timber	Poplar groves, other agricultural wood species
	Rice fields	Areas used forrice cultivation
	Permanent grassland	Permanent grassland without tree and shrub species

## Data Availability

Data is available upon request from the authors; the data that support the findings of this study are available from the corresponding author upon reasonable request.
